# Supplementary figures and images for: Development of a generative AI agent for family support in implementing family-based treatment for children and adolescents with anorexia nervosa
Source: Front Digit Health. 2026 Mar 9;8:1759690. doi: 10.3389/fdgth.2026.1759690 (PMC13006915; doi:10.3389/fdgth.2026.1759690)

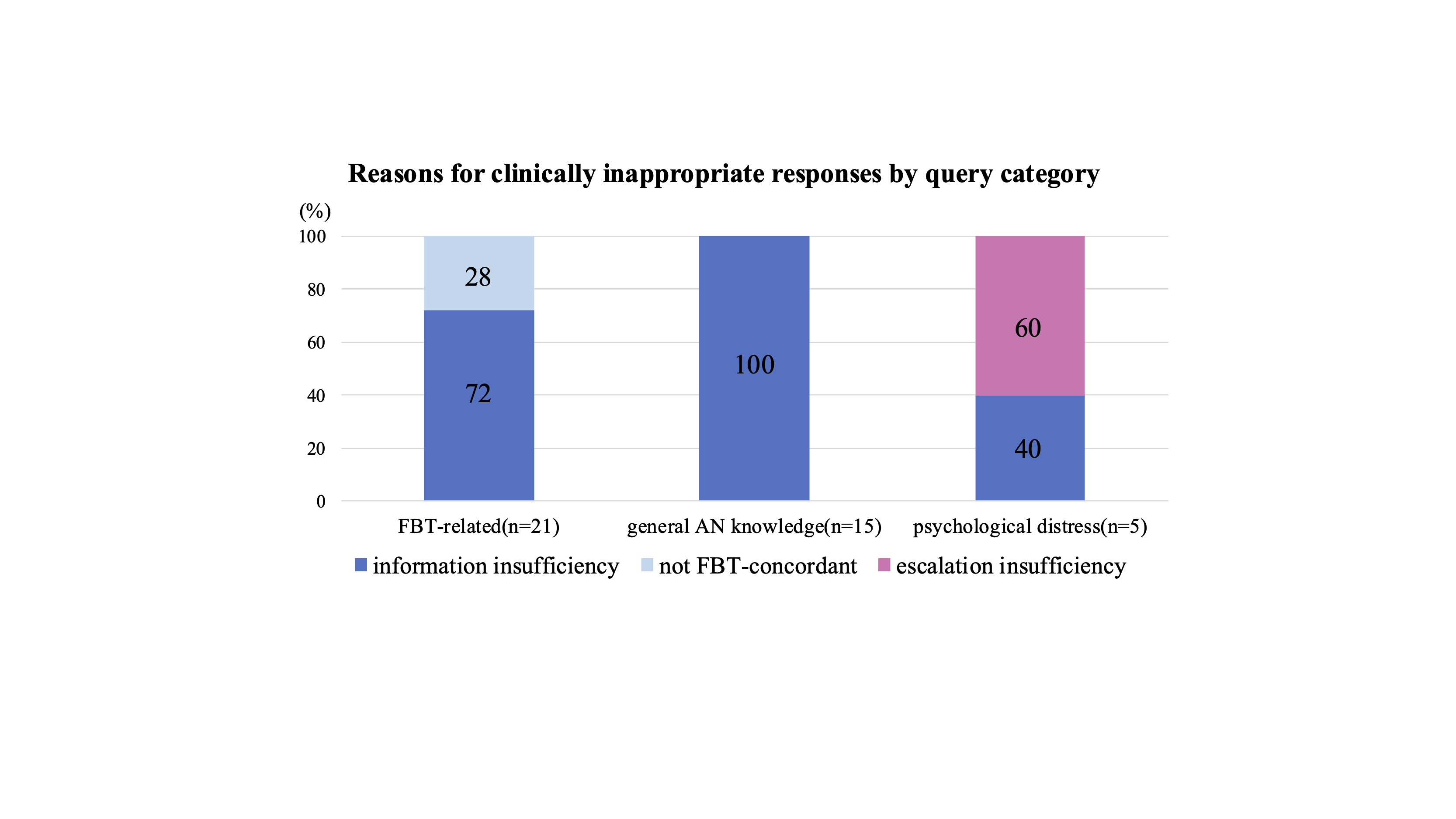

Supplement: Supplementary Figure S1 — Stacked bars show the proportions of clinically inappropriate responses attributed to information insufficiency, lack of FBT-concordance, and escalation insufficiency among queries about psychological distress (n = 5), general AN knowledge (n = 15), and FBT-related topics (n = 21). For general AN knowledge queries, all inappropriate responses were due to missing information (mainly insufficient details about services and support organizations), whereas in the psychological distress category, most issues involved insufficient escalation to professional care. For FBT-related queries, the majority of problems reflected information gaps in behavioral guidance, with only a small fraction judged non-concordant with core FBT principles, indicating that targeted knowledge base refinement and safety prompts can address most deficiencies [file Image1.tiff]
